# Licensing of Yeast Centrosome Duplication Requires Phosphoregulation of Sfi1

**DOI:** 10.1371/journal.pgen.1004666

**Published:** 2014-10-23

**Authors:** Jennifer S. Avena, Shannon Burns, Zulin Yu, Christopher C. Ebmeier, William M. Old, Sue L. Jaspersen, Mark Winey

**Affiliations:** 1 Molecular, Cellular, and Developmental Biology, University of Colorado Boulder, Boulder, Colorado, United States of America; 2 Stowers Institute for Medical Research, Kansas City, Missouri, United States of America; 3 Department of Molecular and Integrative Physiology, University of Kansas Medical Center, Kansas City, Kansas, United States of America; University of California San Francisco, United States of America

## Abstract

Duplication of centrosomes once per cell cycle is essential for bipolar spindle formation and genome maintenance and is controlled in part by cyclin-dependent kinases (Cdks). Our study identifies Sfi1, a conserved component of centrosomes, as the first Cdk substrate required to restrict centrosome duplication to once per cell cycle. We found that reducing Cdk1 phosphorylation by changing Sfi1 phosphorylation sites to nonphosphorylatable residues leads to defects in separation of duplicated spindle pole bodies (SPBs, yeast centrosomes) and to inappropriate SPB reduplication during mitosis. These cells also display defects in bipolar spindle assembly, chromosome segregation, and growth. Our findings lead to a model whereby phosphoregulation of Sfi1 by Cdk1 has the dual function of promoting SPB separation for spindle formation and preventing premature SPB duplication. In addition, we provide evidence that the protein phosphatase Cdc14 has the converse role of activating licensing, likely via dephosphorylation of Sfi1.

## Author Summary

Chromosomes are replicated during the cell cycle and distributed to two progeny cells on a machine called the spindle, such that each cell has one complete copy of the genome. The chromosomes are moved by attachment to the long microtubule polymers of the mitotic spindle, formed from a centrosome at each end of the spindle. Mechanisms that restrict DNA replication and centrosome duplication to once per cell cycle are critical, as defects in either event result in genetic instability and are associated with cancer. Cell cycle-dependent control of DNA replication has been extensively studied, but comparatively little is known about the regulation of centrosome duplication, particularly its restriction to once per cell cycle. Using genetics and cytology, including super-resolution imaging to detect reduplicated centrosomes, we show that cyclin-dependent kinase phosphorylation of Sfi1, a conserved component of centrosomes, prevents the occurrence of extra rounds of yeast centrosome duplication during the cell cycle. Additionally, we propose that dephosphorylation of Sfi1 by the phosphatase Cdc14 permits centrosome duplication for the next cell cycle. Our work is the first to provide a mechanism for how centrosome duplication, like DNA replication, occurs once during the cell cycle through cyclin-dependent kinase regulation.

## Introduction

Centrosomes serve as the poles of the mitotic spindle in eukaryotic cells. Centrosome duplication only once per cell cycle is critical for bipolar spindle organization and proper chromosome segregation. Aberrant centrosome numbers are linked to chromosomal instability and are commonly observed in cancers [1]; thus, mechanisms that restrict duplication to a single event are essential. Cyclin-dependent kinase (Cdk), separase, and polo-like kinase 1 [2]–[5] are known regulators of centrosome duplication implicated in limiting this process to once per cell cycle. However, an understanding of the mechanisms through which they ensure that duplication is tightly coupled with other cell cycle events is still lacking. In budding yeast, deletion of all mitotic cyclins leads to reduplication of spindle pole bodies (SPBs, yeast centrosomes), indicating that mitotic cyclin/Cdk1 blocks SPB reduplication. Thus, a decrease in Cdk1 activity levels is needed to eliminate the block to SPB duplication, allowing SPBs to become licensed, or competent, for duplication [2]. However, even in budding yeast, in which mapping of Cdk1 substrates has been studied at a genome-wide level [6], no targets of Cdk1 that restrict licensing of SPB duplication have been identified.

In budding yeast, the SPB is embedded within the nuclear envelope. Its duplication begins during the G1 phase of the cell cycle when the half-bridge, a specialized region of the nuclear envelope on one side of the SPB, elongates and a density called the satellite forms at the cytoplasmic distal tip. The satellite develops into the new mature SPB, which is linked to the mother SPB via a complete bridge. After duplication is complete and the bridge is severed, the SPBs separate and move to opposite sides of the nucleus as they form the bipolar spindle [7]. All of the components of the SPB have been identified, and several are known to be Cdk1 substrates. In some cases, the role of Cdk1 phosphorylation is known to affect the SPB duplication cycle (Spc42, Sfi1) or spindle properties (γ-tubulin, Spc110) [8]–[14].

Sfi1 is a conserved component of centrosomes and SPBs and is required for SPB duplication in both budding and fission yeast [14]–[17]. Sfi1 is positioned on the cytoplasmic side of the half-bridge in an orientation-specific manner with the N terminus proximal and the C terminus distal to the SPB [18]. Based on this topology, Kilmartin and colleagues put forth a model in which Sfi1 is a central player in both SPB duplication and separation [18]. They proposed that SPB duplication initiates when Sfi1 molecules associate via C-terminal end-to-end interactions with Sfi1 molecules already present at the half-bridge, thus doubling the half-bridge length. A new SPB can then assemble at the free SPB-distal N-terminal domain created by this arrangement. After completion of SPB duplication, dissociation of the Sfi1 C termini at the bridge from each other would then allow SPB separation to occur. Based on this model, it has been proposed that Sfi1 may serve as a licensing factor for SPB duplication [18]–[20]. Interestingly, Sfi1 has been shown to be phosphorylated at numerous sites within C-terminal Cdk1 consensus motifs *in vivo* [11], [21] and *in vitro* by Cdk1 [14] and to be dephosphorylated by the protein phosphatase Cdc14 [14], [20], which is required for mitotic exit [22], [23]. This suggests that Cdk1 and Cdc14 phosphoregulation of Sfi1 contribute to licensing of SPB duplication whereby mitotic Cdk1 phosphorylation of Sfi1 blocks and Cdc14 dephosphorylation enables SPB duplication.

A recent study showed that cells with Sfi1 containing phosphomimetic Cdk1 sites arrest with a single SPB, supporting the idea that phosphorylation of Sfi1 inhibits the process of duplication [14]. However, no studies of Cdk1 targets have mimicked the reduplication phenotype revealed by depletion of mitotic Cdk1 activity [2]. In the current study, we show SPB reduplication in cells with reduced Cdk1 phosphorylation by mutating Sfi1 residues within Cdk1 sites or by activation of Cdc14 in a prolonged anaphase. Our results support the model that Sfi1 C terminus phosphorylation by Cdk1 is required to restrict licensing of SPB duplication and that dephosphorylation of the Sfi1 C terminus by Cdc14 licenses SPBs for duplication.

## Results/Discussion

### Cdk1 phosphorylation of Sfi1 is required for appropriate bipolar spindle assembly and chromosome segregation

Sfi1 contains five residues within full Cdk consensus motifs (S/T*-P-x-K/R), four at the C terminus known to be phosphorylated *in vivo* (T816, S855, S882, S892) [11], [21] and one immediately preceding the C terminus (S801; Fig. S1A). Using a recombinant fragment of Sfi1, we found that four of these residues (S801, T816, S855, S882) and S923 (an *in vivo* phosphorylation site within a minimal Cdk consensus motif (S/T*-P)) are phosphorylated by mitotic cyclin Clb2/Cdk1 *in vitro* (Fig. S1, B–G). *In vitro* Clb2/Cdk1 phosphorylation of three of these residues (T816, S855, S882) as well as S892 has recently been shown [14].

**Figure 1:**
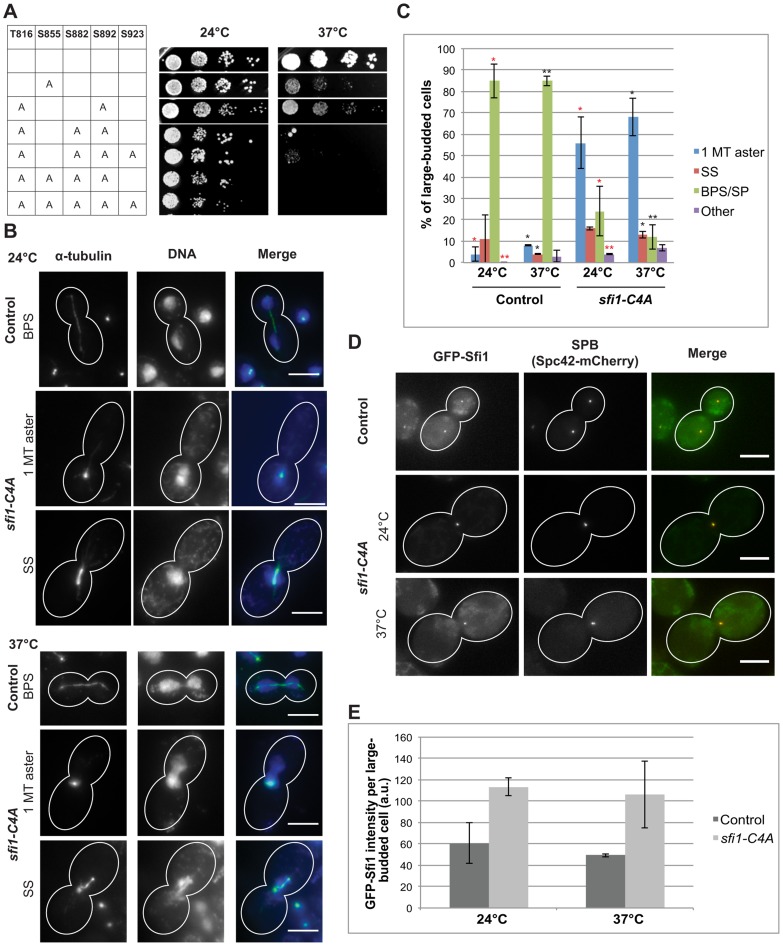
*sfi1-C4A* displays impaired growth and spindle and chromosome segregation defects. **A**. Dilution series of strains with mutations in *SFI1* on YPD. **B–C**. Immunofluorescent staining (α-tubulin: green, DNA: blue) of fixed large-budded *sfi1-C4A* (JA249) or control (JA196) cells grown at 24°C or shifted to 37°C for 8.25 h in YPD. MT: microtubule, SS: short metaphase bipolar spindle, BPS/SP: bipolar spindle or separated poles. Bar: 5 µm. **C**. Quantification of B. Asterisks: statistically significant difference via Student's *t* test between *sfi1-C4A* and control for each phenotypic category at 24°C (red) and 37°C (black). **p<0.01. *: p<0.05. Significance only shown for comparisons between strains at each temperature. Error bars: SD. n≥192 cells per group from 2 experiments. **D–E**. *GFP-sfi1-C4A pLEU-HIS-SPC42-mCherry* (JA354) or *GFP-SFI1 pLEU-HIS-SPC42-mCherry* (JA311) grown in SC-Leu at 24°C or shifted to 37°C 9 h were fixed prior to localization (D) of Sfi1 (green) and Spc42 (red) and quantification of Sfi1 levels in arbitrary units (a.u.) (E). Error bars: SD. n≥62 cells per group from 2 experiments. Bar: 5 µm.

We created a nonphosphorylatable mutant allele collection with individual and combinations of these C-terminal residues converted to alanine. Alteration of S855 resulted in a temperature-sensitive growth defect at 37°C (Fig. 1A). Combinations of mutations resulted in varying phenotypes. We found that the alleles *sfi1-C3A* (T816A S882A S892A) and *sfi1-C4A* (T816A S855A S882A S892A) show growth defects at 24°C and little or no growth, respectively, at 37°C (Fig. 1A). Mutation of the minimal Cdk consensus site (S923A) in combination with *sfi1-C3A* or *sfi1-C4A* does not enhance the growth defect (Fig. 1A).

The *sfi1-C4A* and *sfi1-C3A* mutant proteins fused to GFP localize to the SPB at levels similar or greater than that of GFP-Sfi1 (Fig. 1, D and E, and Fig. S2, C and D, respectively), indicating that the phenotypes observed are not caused by reduced levels of the mutant proteins but rather are likely due to loss of Cdk1 phosphorylation. In an asynchronous population of *sfi1-C4A* or *sfi1-C3A*, large-budded cells displayed multiple spindle morphology phenotypes (Fig. 1, B and C and Fig. S2, A and B, respectively). *sfi1-C4A* and *sfi1-C3A* displayed significant increases both in short spindles and in single microtubule asters with chromosome missegregation at 37°C compared to control. Defects were also observed at 24°C.

### Cdk1 phosphorylation of Sfi1 is required for SPB separation and to block SPB reduplication

**Figure 2:**
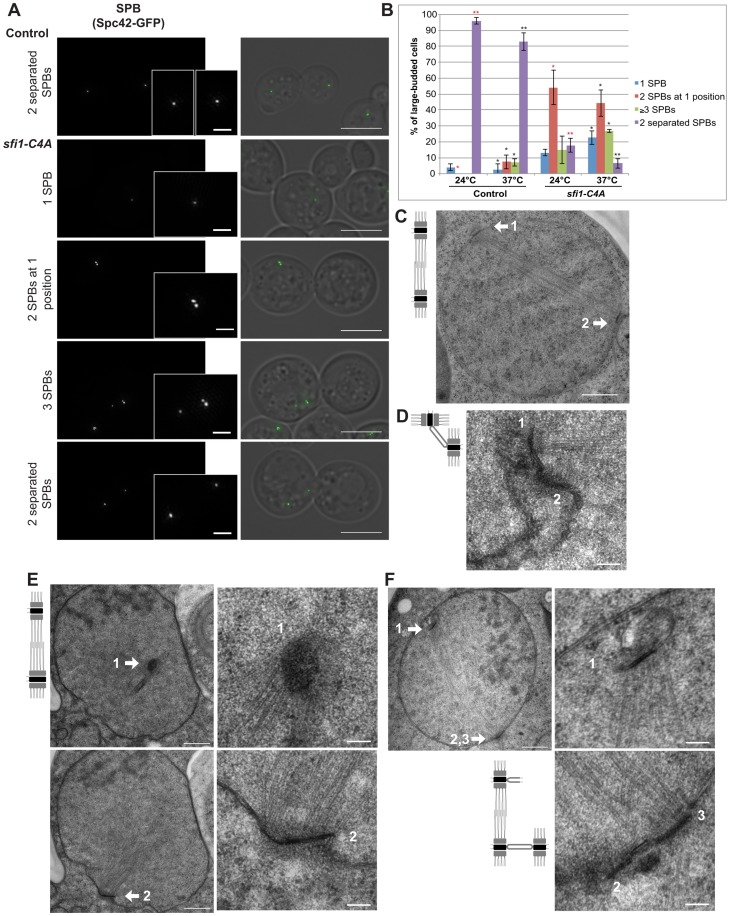
*sfi1-C4A* displays unseparated SPBs or bipolar spindles with enlarged or reduplicated SPBs. **A**. Asynchronous *sfi1-C4A SPC42-GFP* (JA302) and *SFI1 SPC42-GFP* (JA254) control cells grown at 24°C and shifted to 37°C for 9 h in YPD. Fixed large-budded cells were imaged by SIM. GFP on left (inset bar: 1 µm) and merge with transmitted image on right. Bar: 5 µm. **B**. Quantification of A. Asterisks: statistically significant difference via Student's *t* test between *sfi1-C4A* and control for each phenotypic category at 24°C (red) and 37°C (black). **p<0.01. *: p<0.05. Significance only shown for comparisons between strains at each temperature. Error bars: SD. n≥77 cells per group from 2 experiments. **C**. Asynchronous control cells (JA196) shifted to 37°C for 4 h in YPD and prepared for EM. Bipolar spindle with two SPBs (1, 2). Bar: 500 nm. **D–F**. Asynchronous *sfi1-C4A* (JA249) cells shifted to 37°C for 9 h in YPD and prepared for EM. Serial sections were examined by EM for 18 cells. **D**. Representative cell (n = 11) with two SPBs at one position. SPBs (1, 2) are at abnormal orientations to one another with a bridge. Bar: 100 nm. **E–F**. Multiple sections from the same cell are shown. Left panel(s): entire nucleus, 500 nm bar. Right panels: SPB(s), 100 nm bar. **E**. Representative cell (n = 2) containing a short bipolar spindle with SPB 1 at an orientation different from that of abnormally large SPB 2. **F**. Representative cell (n = 5) containing a short bipolar spindle with extra SPBs. SPB 1 is located at a region of the nuclear envelope opposite SPBs 2 and 3, which are connected via a bridge. Left panel shows the location of SPB 1, shown in the upper right panel at higher magnification, and SPBs 2 and 3. In the lower right panel, both SPBs 2 and 3 are clearly seen in a higher magnification view from a different serial section of the same cell. Average distance between adjacent SPBs: 108±10 nm.

The phenotypes of *sfi1-C4A* in large-budded cells were examined using structured illumination microscopy (SIM; Fig. 2A). We used Spc42-GFP to label both the SPB and the assembly intermediate, the satellite [24]. SIM allowed us to resolve side-by-side SPBs and a satellite versus mother SPB into two Spc42-GFP foci. In contrast to control cells containing mainly two separated SPBs, we found that *sfi1-C4A* cells at 37°C commonly displayed one of the following three phenotypes: a single (Spc42-GFP) focus, two adjacent foci suggestive of side-by-side SPBs, or at least three foci (Fig. 2, A and B). Commonly, in *sfi1-C4A* large-budded cells that contained at least three GFP foci, two foci were located adjacent to one another, while a third GFP focus was located at a distance (13.71±6.92% at 24°C, 22.57±1.18% at 37°C; Fig. 2A). The observation of more than two SPBs suggests that an aberrant reduplication event has occurred.

Using electron microscopy (EM), we further studied the phenotype of *sfi1* mutant cells. Control large-budded cells display a bipolar spindle with two SPBs, one at either side of the nucleus (Fig. 2C). In *sfi1-C4A* large-budded cells, we observed either unseparated SPBs at the same position within the nuclear envelope (n = 11 of 18 cells), suggesting a defect in SPB separation (Fig. 2D), or short bipolar spindles with aberrant SPBs (n = 7 of 18 cells, Fig. 2, E and F). Some cells with short spindles have two SPBs, with one being enlarged (n = 2; Fig. 2E). However, the remaining cells (n = 5) contain three or four SPBs, in which two SPBs are adjacent, and the other SPB (or two adjacent SPBs when there are four total) is connected by the short spindle and lies on the other side of the nuclear envelope (Fig. 2F). Given that we typically see the presence of a bridge connecting two adjacent SPBs in these cells (n = 4 of 5 cells, Fig. 2F), and the distance between these SPBs is approximately that expected in wild type cells (Fig. 2F) [18], [25], we conclude that an aberrant reduplication event has occurred. A similar phenotype was also observed in *sfi1-C3A* (Fig. S3). This data is consistent with our SIM results and indicates that loss of Cdk1 phosphorylation of Sfi1 leads to SPB reduplication. Specifically, in cells with reduplicated SPBs, as seen previously in cells in which all mitotic cyclins are deleted [2], each extra SPB has duplicated using one of the original SPBs present in the spindle as a “template” during a single cell cycle. Thus, the nonphosphorylatable *sfi1* mutations lead to premature licensing of SPB duplication during mitosis. Excitingly, this now points to Sfi1 as the first target of Cdk1 in blocking reduplication.

**Figure 3:**
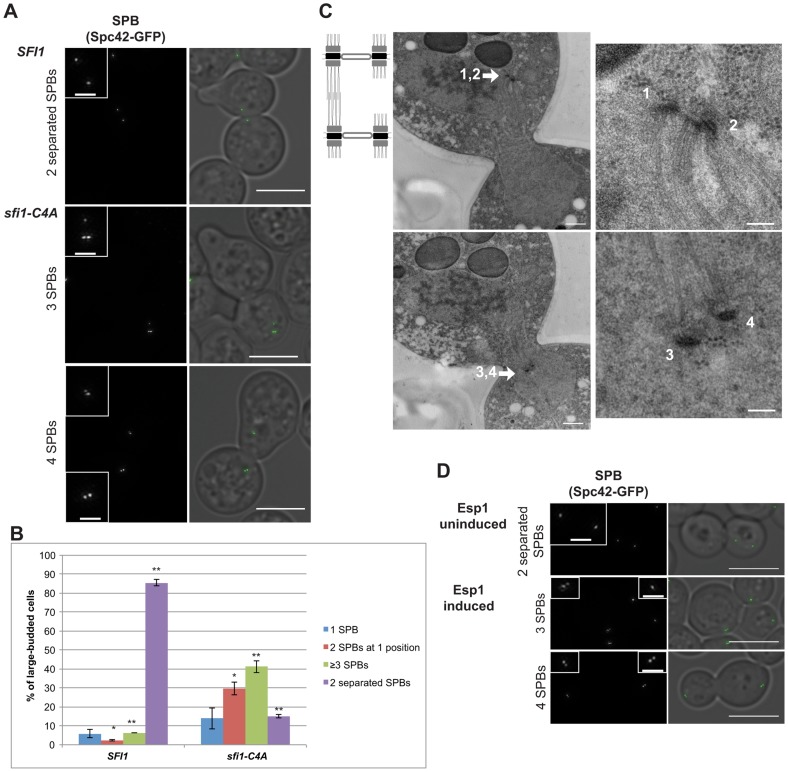
Reduplicated SPBs upon mitotic arrest are enhanced in *sfi1-C4A* and are present upon Cdc14 activation. **A–C**. *SFI1* (JA295) or *sfi1-C4A* (JA297) cells containing *GAL1-pds1-mdb SPC42-GFP* were grown to early-log phase at 24°C in YEP with 2% raffinose then arrested in G1 with α-factor (≤2 SPBs at G1 arrest: 98.1±0.4% for *sfi1-C4A*, 99.4±0.4% for *SFI1*; n≥219 per group from 2 experiments). G1-arrested cells were released into YEP with 2% galactose to arrest in mitosis and, once at mitotic arrest, were shifted to 37°C for 2.5 h. **A**. Fixed large-budded cells were imaged by SIM, with GFP on left (inset bar: 1 µm) and merge with transmitted image on right. Bar: 5 µm. Mitotically-arrested cells following 37°C shift. **B**. Quantification of A. Asterisks: statistically significant difference via Student's *t* test. **p<0.01. *: p<0.05. Error bars: SD. n≥185 cells per group from 2 experiments. **C**. A *sfi1-C4A GAL1-pds1-mdb SPC42-GFP* cell imaged by EM containing at least four SPBs (1–4) total. Left panels: entire nucleus, 500 nm bar. Right: SPBs, 100 nm bar. **D.** An asynchronous culture of *MET-CDC20 cdh1Δ GALS-ESP1 SPC42-GFP* (JA256) cells was grown to early-log phase in SC-Met with 3% raffinose. Methionine was added (2 mM final concentration) to arrest cells in metaphase and every 2 h for the experiment remainder. After metaphase arrest, Esp1 either was induced for 4 h using 3% galactose to release Cdc14 from the nucleolus or remained uninduced in raffinose. Fixed large-budded cells were imaged by SIM, with GFP on left (inset bar: 1 µm) and merge with transmitted image on right. Bar: 5 µm. n≥211 per group from 2 experiments. Upper panel: cells with no Esp1 induction with bipolar spindles (63±13%). Lower panels: cells with Esp1 induction with one or two reduplicated SPBs (3 or 4 Spc42-GFP foci total, respectively; 22±1%); p = 0.02 (Student's *t* test) for reduplicated SPBs versus without Esp1 induction (7±3%).

Since we propose that the aberrant SPB reduplication events observed in *sfi1-C4A* occur during mitosis within a single cell cycle, we would expect that mitotically-arrested *sfi1-C4A* cells would display reduplicated SPBs. Therefore, we arrested cells in mitosis by inducing overexpression of a nondegradable Pds1 (*GAL1-pds1-mdb*) [26] and then shifted to 37°C. The cells were initially arrested in G1, in which both *SFI1* and *sfi1-C4A GAL1-pds1-mdb* cells commonly had two Spc42-GFP foci (>98%, see Fig. 3 legend), indicative of a mother SPB and satellite. As expected, *SFI1 GAL1-pds1-mdb* cells released from G1 and arrested in mitosis at 37°C displayed two separated (Spc42-GFP) foci, indicative of a bipolar spindle (86±2%; Fig. 3, A and B). In contrast, *sfi1-C4A GAL1-pds1-mdb* cells arrested in mitosis at 37°C only occasionally displayed two separated foci (15±1%). *sfi1-C4A GAL1-pds1-mdb* cells sometimes showed two foci at one position (30±3%), indicating that SPB duplication occurred, but not separation, but predominantly displayed at least three foci (41±3%; Fig. 3, A and B). A majority of *sfi1-C4A* cells with at least three Spc42-GFP foci contained at least two adjacent foci with additional SPB(s), indicative of reduplicated SPBs (36.8±1.4%; Fig. 3A). We confirmed the presence of reduplicated SPBs via EM (Fig. 3C). These findings support our conclusion that SPB reduplication within a single cell cycle is responsible for the presence of additional SPBs in cells lacking Cdk1 phosphorylation sites in Sfi1.

### Cdc14 is required to license SPB duplication

Cdk1 activity is counteracted by a phosphatase, Cdc14, which is activated during anaphase. Cdc14 is thought to remove Cdk1-dependent phosphates from a number of targets [22], [23] and has been shown to dephosphorylate Sfi1 [14], [20]. Several Cdk sites on Sfi1 are expected to be good Cdc14 target sites, as they are within Cdc14 consensus motifs (S-P-x-K/R; S801, S855, S882, S892) [14], [27], [28]. To determine whether Cdc14 acted on Sfi1 to control licensing of SPB duplication, we utilized an experimental strategy previously used by Cross and colleagues to examine another Cdc14-regulated dephosphorylation event in mitosis [20], [29]. Specifically, *MET-CDC20 cdh1Δ GALS-ESP1* cells arrested in metaphase via repression of *CDC20* were driven into anaphase by induction of separase (Esp1), which leads to Cdc14 release from the nucleolus and activation. Lack of both Cdc20 and Cdh1 prevents mitotic exit into G1 [20], [29], [30]. This engineered strain, both with and without Esp1 induction, includes a population of large-budded cells with three or more dispersed Spc42-GFP foci, which may arise from SPB missegregation events (17±7% and 25±8%, respectively; Fig. S4). However, notably, significantly more actual SPB reduplication events (two adjacent SPBs with additional SPBs in large-budded cells, 22±1%) were seen in cells in which Esp1 overexpression was induced and Cdc14 was activated than in cells in which Esp1 was not induced (7±3%; Fig. 3D), indicating that active Cdc14 promotes the presence of reduplicated SPBs.

A recent study showed that *cdc14-2* cells forced into G1 were delayed in SPB duplication [14]. The authors conclude that Cdc14 promotes timely SPB duplication, which is consistent with our work demonstrating that Cdc14 is specifically involved in licensing of SPB duplication, as evidenced by the presence of reduplicated SPBs with prolonged activation of Cdc14. Schiebel and colleagues did not observe an increase in Sfi1 incorporation into the SPB, as measured by Sfi1 fluorescent signal intensity, with overexpression of Cdc14 in a metaphase arrest [14]. Using the protocol from the Cross lab [20], [29], we were able to arrest cells at a stage in the cell cycle that was permissive for Cdc14-driven reduplication, and we directly examined SPB assembly using SIM. While Cln/Cdk1 activity is required for SPB duplication in G1 [31], in mitotically-arrested cells in the absence of mitotic cyclin degradation [32]–[35], it appears that mitotic cyclins permit SPB duplication when Sfi1 phosphorylation is diminished.

**Figure 4:**
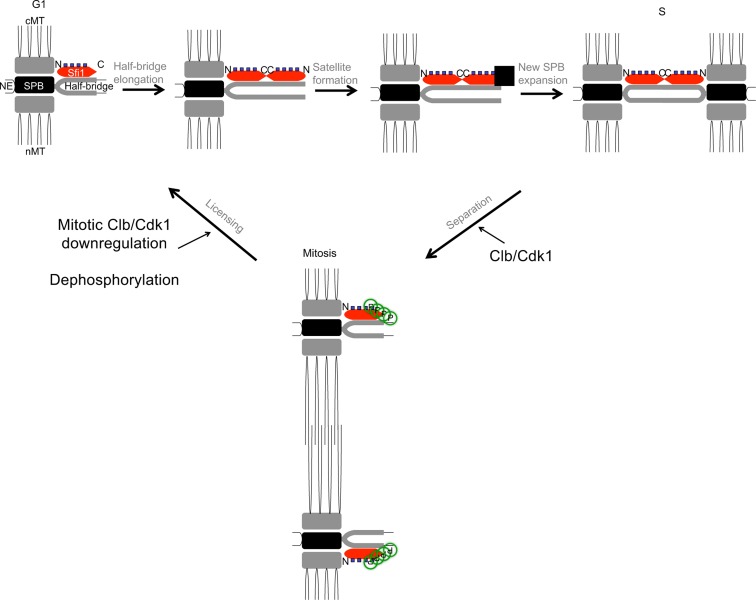
Model for licensing of yeast centrosome duplication via phosphoregulation of Sfi1. Cdk1 activity is required for SPB separation, in which Cdk1 phosphorylates the Sfi1 C terminus, ensuring SPB duplication does not begin until completion of mitosis and downregulation of mitotic cyclin/Cdk1. Dephosphorylation of Sfi1, likely by Cdc14, licenses SPB duplication to allow the next cycle of SPB duplication to begin at G1. cMT: cytoplasmic microtubules. nMT: nuclear microtubules. NE: nuclear envelope. ▪: satellite.

Our findings support the model that Cdk1 phosphorylation of Sfi1 is required for SPB separation and a block to SPB reduplication (Fig. 4). Specifically, we propose that after SPB duplication is complete, Cdk1 phosphorylates at least four Sfi1 C-terminal residues, allowing for SPB separation and bipolar spindle formation during mitosis. These C-terminal residues remain phosphorylated during mitosis, restricting licensing of SPB duplication until downregulation of mitotic cyclin/Cdk1 at the end of mitosis. Dephosphorylation of Sfi1 by Cdc14 at the end of mitosis then licenses the SPBs for the next cycle of G1 SPB duplication.

Consistent with our findings, previous research has shown SPB separation requires mitotic cyclin/Cdk1 [2], [36], [37] and Cdk1 phosphorylation of the Sfi1 C terminus [14], [38]. In addition, Schiebel and colleagues showed that cells with Sfi1 containing six phosphomimetic Cdk1 sites arrest with a single SPB, indicating that Cdk1 phosphorylation of Sfi1 inhibits SPB duplication [14]. This result supports our conclusion that the block to SPB duplication by Cdk1 phosphorylation of Sfi1 must be removed to license SPB duplication. Importantly, we have reproduced the reduplication phenotype seen upon depletion of mitotic Cdk1 activity [2], either by nonphosphorylatable versions of Sfi1 or by inducing Cdc14 activity. Appropriate phosphoregulation of Sfi1 by Cdk1 and Cdc14 thus ensures SPB duplication occurs only once per cell cycle.

Future work examining whether the Sfi1 C termini directly interact with each other and/or interact with other components of the SPB and the impact of phosphorylation status on these interactions will be important in understanding the mechanism that initiates SPB duplication. Moreover, further studies will prove useful in determining a role for Cdks in licensing in metazoans. Given that human Sfi1 also contains multiple Cdk consensus motifs at the C terminus, this study could provide crucial insight into the licensing of centrosome duplication.

## Materials and Methods

### Yeast strains

The W303 strain background was used for all experiments (Table S1). Yeast transformation with pRS402 was used to create *ADE2* strains where indicated in the strain list. Standard PCR mutagenesis, including use of the Quikchange II mutagenesis kit (Agilent), was used to create all mutations within the *SFI1* sequence using a pRS305 vector base. Control and mutant strains (except JA256 and JA295) contain a silent mutation at Sfi1 residues E710 and L711 for a nucleotide change of A2130G T2131C in order to develop an additional restriction enzyme site within the Sfi1 sequence for plasmid manipulation purposes. Creation of single integrant mutants with the NATMX marker via PCR is as previously described [39]. This technique was used to create N-terminally tagged GFP-Sfi1 strains by transforming *trp1::N-GFP-SFI1-TRP1 sfi1Δ::HIS5* (gift of John Kilmartin) [16]. Strains JA295 and JA297 were created by integrating pOC70 (gift of Orna Cohen-Fix) [26] at the *LEU2* locus of heterozygous diploid strains, followed by dissection and selection of appropriate haploids. C-terminal tagging of *SPC42* with *yeGFP1* via PCR was performed as previously described [40], [41]. JA256 was created using YL165 (gift of Fred Cross) [29] via mating.

Five-fold dilution series with 5 µL of cells at an initial OD/mL of 0.1 in the left column were placed on YPD plates and grown at the indicated temperatures for 2 days. For temperature shift experiments of asynchronous cultures, cultures were shifted at early-log phase.

### Cytological techniques

Imaging of live and fixed cells was performed at room temperature on an Eclipse Ti inverted microscope (Nikon, Tokyo, Japan) fitted with a CFI Plan Apo VC 60× H numerical aperture 1.4 objective (Nikon, Tokyo, Japan) and a CoolSNAP HQ2 charge-coupled device camera (Photometrics, Tuscon, AZ, USA). Metamorph imaging software (Molecular Devices, Sunnyvale, CA, USA) was used to collect images, and maximum projections are shown. The 1.5× intermediate magnification was used for fixed immunofluorescent images.

For immunofluorescence, cultures were fixed with 4% formaldehyde for 45 minutes, subjected to zymolyase and prepared on slides. YOL1/34 rat anti-tubulin antibody (1/150), FITC goat anti-rat secondary antibody (1/200), and Hoechst dye for DNA were used. For imaging of live GFP-expressing cells, cells were briefly centrifuged, washed, and imaged in 1× PBS. For brief fixation of GFP-expressing cells, cultures were fixed with 3.7% formaldehyde for 15 minutes, resuspended, and imaged in 1×PBS or KPO_4_/sorbitol.

SIM images were acquired at room temperature on an Applied Precision OMX Blaze microscope (Issaquah, WA, USA) equipped with a PCO Edge sCMOS camera (Kelheim, Germany). The objective used was an Olympus (Center Valley, PA, USA) 60× 1.42NA Plan Apo N oil objective. Image stacks were acquired at 125 nm intervals. SIM reconstruction was performed with the Applied Precision software package utilizing optical transfer functions measured with 100 nm green fluospheres in Prolong Gold on a coverslip surface (Life Technologies, Carlsbad, CA, USA) following the Applied Precision protocols. After reconstruction, SIM images were scaled 2 by 2 with bilinear interpolation through ImageJ software (National Institutes of Health, Bethesda, MD, USA) for future quantification.

Quantification of GFP intensities in live and fixed GFP-expressing cells was performed with Metamorph imaging software using maximum projections, in which the average pixel intensity was determined for the fluorescent focus and subtracted from the average pixel intensity for the immediate background border. Distances were measured using ImageJ software. GFP intensity (arbitrary units; a.u.) was averaged for each strain at each temperature, and the average of this value from two experiments for each condition was taken. A Student's *t* test was then performed on the average from both experiments.

### Transmission electron microscopy

Log phase cells were high pressure frozen in a Wohlwend Compact 02 HPF and freeze-substituted in 2% osmium tetroxide, 0.1% uranyl acetate in acetone and embedded in Spurr's epoxy (JA188) or in Epon (JA196) or freeze-substituted in 0.25% glutaraldehyde, 0.1% uranyl acetate in acetone and embedded in Lowicryl HM20 (JA249, JA297) [42]. Imaging was conducted using a FEI Phillips CM100 electron microscope.

Distances in electron micrographs were measured using ImageJ software. Enlarged SPBs were classified as >213.28 nm diameter, which is >33.33% deviance from the predicted average diameter of 160 nm in a diploid [31].

### Protein techniques and kinase reaction

Sfi1 residues 800 to 946 were amplified via PCR and cloned into the pKLD116 plasmid (gift of Ivan Rayment) immediately downstream of the rTEV site and in frame with the N-terminal 6×His and maltose binding protein tags. The resulting plasmid was transformed into C+ (DE3) competent *E. coli* cells (gift of Greg Odorizzi). IPTG induction was performed using 0.3 mM IPTG for 2.5 h at 23°C. The sample was flash frozen, treated with lysozyme, sonicated, and column purification using Talon metal affinity resin (Clontech Laboratories, Inc.) was performed using 200 mM imidazole for elution. An *in vitro* kinase reaction was performed using 1 µg of the Sfi1 C terminus fusion protein (6×His-MBP-rTEVsite-Sfi1C), 1 mM ATP, and Clb2 (purified from bacteria)/Cdc28 (purified from baculovirus co-infected with the Cdk activating kinase (CAK)) [8].

### Mass spectrometry

The 20 µL kinase reaction was digested with 2 µg GluC (no denaturation nor reduction/alkylation) and incubated at room temperature for 1 h. 2 µL or 10% of the reaction was loaded directly onto a Waters nanoAcquity 75 µm×250 mm 1.7 µm BEH130 C18 column (no trapping nor desalting). Peptides were eluted with a gradient from 8% acetonitrile, 0.1% formic acid to 32% acetonitrile, 0.1% formic acid at a flow rate of 300 nL/min. Spectra were searched using Mascot v2.2 (Matrix Science) against a small custom database with the protein sequence of the recombinant Sfi1 fusion protein included. Phosphorylated peptides were identified by manual inspection of all MS/MS spectra with Mascot ions scores of at least 20. Multiple phosphorylated isoforms were identified as possible if the delta score was less than 4 (difference between the top two scoring phosphorylated positional isomers).

## Supporting Information

Figure S1 Clb2/Cdk1 phosphorylates Sfi1 at sites within Cdk motifs *in vitro*. **A.** Sfi1 schematic with N-terminal, Sfi1 repeat, and C-terminal domains. The Sfi1 repeat domain contains 21 conserved Sfi1 repeat sequences [16], [18]. The C terminus is defined as all residues (802–946) immediately following the final Sfi1 repeat sequence [38]. All residues within full Cdk1 consensus motifs and the single known phosphorylated residue within a C-terminal minimal Cdk1 consensus motif (S923) are identified. **B.** Identification of phosphorylated residues from two replicate mass spectrometry runs of *in vitro* phosphorylated recombinant Sfi1 C terminus fusion protein by Clb2/Cdk1. Coverage of Sfi1 was 88% for both runs. If multiple phosphorylated isoforms are probable, highest ions score is in bold, with brackets identifying the region of sites potentially phosphorylated. Sites previously identified *in vivo* are indicated [11], [21]. pS/T: phosphorylated residue. S/T(P): Cdk site. **C–G.** Annotated spectra with defining ions only of *in vitro* phosphorylated residues within full Cdk1 consensus and minimal C-terminal consensus motifs. All identified b and y ions, summarized for all ions with or without neutral, water, and/or ammonia loss at any charge state, are indicated for each peptide sequence, with the phosphorylated residue in red as “pS/T.” –H_3_PO_4_: neutral loss, -H_2_0: water loss, -NH_3_: ammonia loss. **C.** S801. Parent *m/z* 848.76; z = 3. **D.** T816. Parent *m/z* 1041.00; z = 2. **E.** S855. Parent *m/z* 768.40; z = 2. **F.** S882. Parent *m/z* 742.38; z = 3. Ion y13^+^ –H_3_PO_4_ -NH_3_ at *m/z* 1480.19. **G.** S923. Parent *m/z* 780.39; z = 3.
(TIF)

Figure S2 *sfi1-C3A* displays spindle and chromosome segregation defects. **A–B**. Immunofluorescent staining (α-tubulin: green, DNA: blue) of fixed large-budded *sfi1-C3A* (JA188) or control (JA196) cells grown at 24°C or shifted at early-log phase to 37°C for 4 h in YPD. MT: microtubule, SS: short metaphase bipolar spindle, BPS/SP: bipolar spindle or separated poles. Bar: 5 µm. **B**. Quantification of A. Asterisks indicate a statistically significant difference using the Student's *t* test between *sfi1-C3A* and control for each phenotypic category at 24°C (red) and 37°C (black). **p<0.01. *: p<0.05. Significance is only shown for comparisons between strains at each temperature. Error bars: SD. n≥200 cells per group from 2 experiments. **C–D**. Asynchronous cultures *GFP-sfi1-C3A pLEU-HIS-SPC42-mCherry* (JA308, JA309) or *GFP-SFI1 pLEU-HIS-Spc42-mCherry* (JA310, JA311) were grown at 24°C in SC-Leu and shifted at early-log phase to 37°C for 4 h. Cells were briefly fixed prior to imaging or imaged live. **C.** Localization of GFP-Sfi1 (green) and Spc42-mCherry (red) and a merged image for fixed control at 37°C and *sfi1-C3A* cells at 24°C and 37°C. Bar: 5 µm. **D.** Quantification of C. *p<0.05 via Student's *t* test. Error bars: SD. n≥14 cells per group from 2 experiments.
(TIF)

Figure S3 *sfi1-C3A* displays unseparated SPBs or bipolar spindles with enlarged or reduplicated SPBs. **A–C**. Asynchronous *sfi1-C3A* (JA188) cells shifted at early-log phase to 37°C for 4 h in YPD were prepared for EM. Serial sections were examined for 21 cells. **A**. Representative cells containing two SPBs at one pole with SPBs in a side-by-side configuration (n = 8; upper panel) or SPBs with an abnormal orientation (n = 7; lower panel). Scale bar: 100 nm. **B–C**. Cells contain short bipolar spindles with aberrant SPBs. Left panels: full spindle, 500 nm scale bar. Right panels: SPB(s), 100 nm scale bar. **B**. Representative cell (n = 3) with an abnormally large SPB in the upper panel. **C**. Representative cell (n = 3) containing a short bipolar spindle with three SPBs. The upper panel identifies a single SPB (1), while the lower panel shows a mature SPB (3) connected via a bridge to a partial SPB (2). Average distance between adjacent SPBs: 148±22 nm.
(TIF)

Figure S4 A portion of *MET-CDC20 cdh1Δ GALS-ESP1* cells displays at least three dispersed Spc42-GFP foci. An asynchronous culture of *MET-CDC20 cdh1Δ GALS-ESP1 SPC42-GFP* (JA256) cells was grown to early-log phase in SC-Met with 3% raffinose. Methionine was added (2 mM final concentration) to arrest cells in metaphase and every 2 h for the experiment remainder. After metaphase arrest, Esp1 either was induced for 4 h using 3% galactose to release Cdc14 from the nucleolus or remained uninduced in raffinose. Fixed large-budded cells were imaged by SIM, with GFP on left and merge with transmitted image on right. A portion of cells, both without (25±8%, upper panel) and with (17±7%, lower panel) Esp1 induction, contain at least three dispersed Spc42-GFP foci. Bar: 5 µm. n≥211 per group from 2 experiments.
(TIF)

Table S1 Yeast strains. Yeast strains used in this study.
(DOC)
