# Supplementary figures and images for: Licensing of Yeast Centrosome Duplication Requires Phosphoregulation of Sfi1
Source: PLoS Genet. 2014 Oct 23;10(10):e1004666. doi: 10.1371/journal.pgen.1004666 (PMC4207612; doi:10.1371/journal.pgen.1004666)

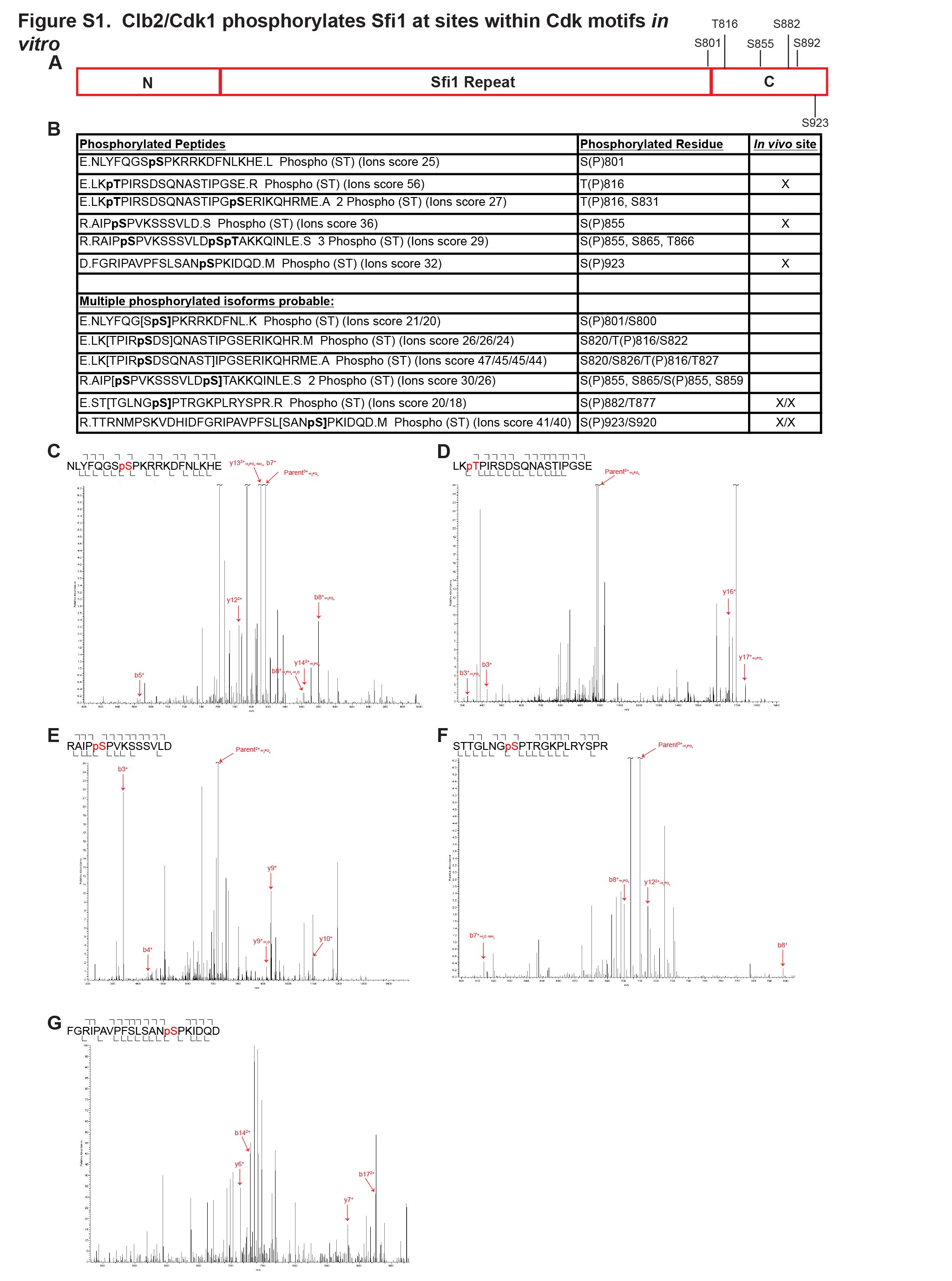

Supplement: Figure S1 — Clb2/Cdk1 phosphorylates Sfi1 at sites within Cdk motifs in vitro. A. Sfi1 schematic with N-terminal, Sfi1 repeat, and C-terminal domains. The Sfi1 repeat domain contains 21 conserved Sfi1 repeat sequences [16], [18]. The C terminus is defined as all residues (802–946) immediately following the final Sfi1 repeat sequence [38]. All residues within full Cdk1 consensus motifs and the single known phosphorylated residue within a C-terminal minimal Cdk1 consensus motif (S923) are identified. B. Identification of phosphorylated residues from two replicate mass spectrometry runs of in vitro phosphorylated recombinant Sfi1 C terminus fusion protein by Clb2/Cdk1. Coverage of Sfi1 was 88% for both runs. If multiple phosphorylated isoforms are probable, highest ions score is in bold, with brackets identifying the region of sites potentially phosphorylated. Sites previously identified in vivo are indicated [11], [21]. pS/T: phosphorylated residue. S/T(P): Cdk site. C–G. Annotated spectra with defining ions only of in vitro phosphorylated residues within full Cdk1 consensus and minimal C-terminal consensus motifs. All identified b and y ions, summarized for all ions with or without neutral, water, and/or ammonia loss at any charge state, are indicated for each peptide sequence, with the phosphorylated residue in red as “pS/T.” –H3PO4: neutral loss, -H20: water loss, -NH3: ammonia loss. C. S801. Parent m/z 848.76; z = 3. D. T816. Parent m/z 1041.00; z = 2. E. S855. Parent m/z 768.40; z = 2. F. S882. Parent m/z 742.38; z = 3. Ion y13+ –H3PO4 -NH3 at m/z 1480.19. G. S923. Parent m/z 780.39; z = 3. (TIF) [file pgen.1004666.s001.tif]

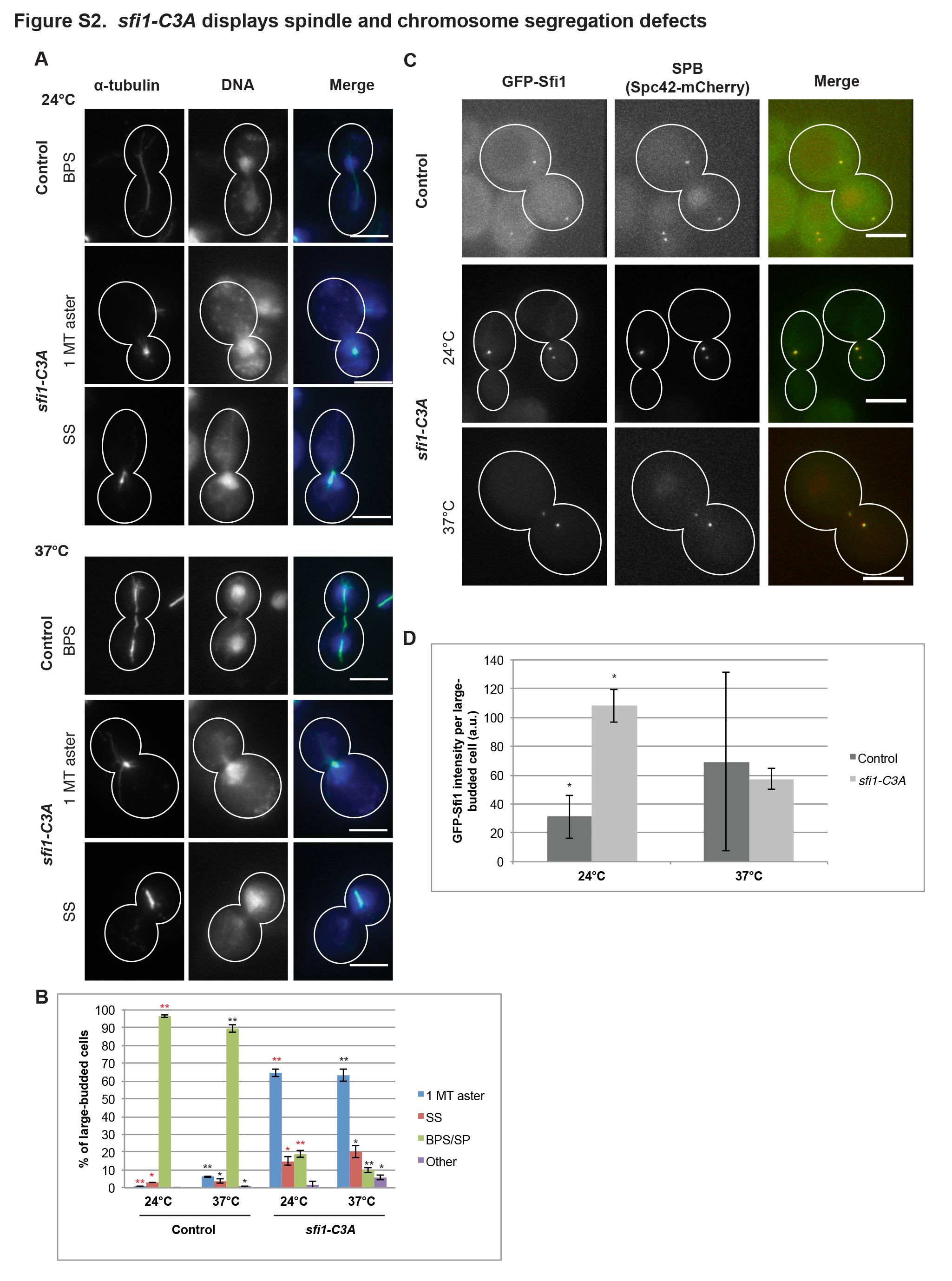

Supplement: Figure S2 — sfi1-C3A displays spindle and chromosome segregation defects. A–B. Immunofluorescent staining (α-tubulin: green, DNA: blue) of fixed large-budded sfi1-C3A (JA188) or control (JA196) cells grown at 24°C or shifted at early-log phase to 37°C for 4 h in YPD. MT: microtubule, SS: short metaphase bipolar spindle, BPS/SP: bipolar spindle or separated poles. Bar: 5 µm. B. Quantification of A. Asterisks indicate a statistically significant difference using the Student's t test between sfi1-C3A and control for each phenotypic category at 24°C (red) and 37°C (black). **p<0.01. *: p<0.05. Significance is only shown for comparisons between strains at each temperature. Error bars: SD. n≥200 cells per group from 2 experiments. C–D. Asynchronous cultures GFP-sfi1-C3A pLEU-HIS-SPC42-mCherry (JA308, JA309) or GFP-SFI1 pLEU-HIS-Spc42-mCherry (JA310, JA311) were grown at 24°C in SC-Leu and shifted at early-log phase to 37°C for 4 h. Cells were briefly fixed prior to imaging or imaged live. C. Localization of GFP-Sfi1 (green) and Spc42-mCherry (red) and a merged image for fixed control at 37°C and sfi1-C3A cells at 24°C and 37°C. Bar: 5 µm. D. Quantification of C. *p<0.05 via Student's t test. Error bars: SD. n≥14 cells per group from 2 experiments. (TIF) [file pgen.1004666.s002.tif]

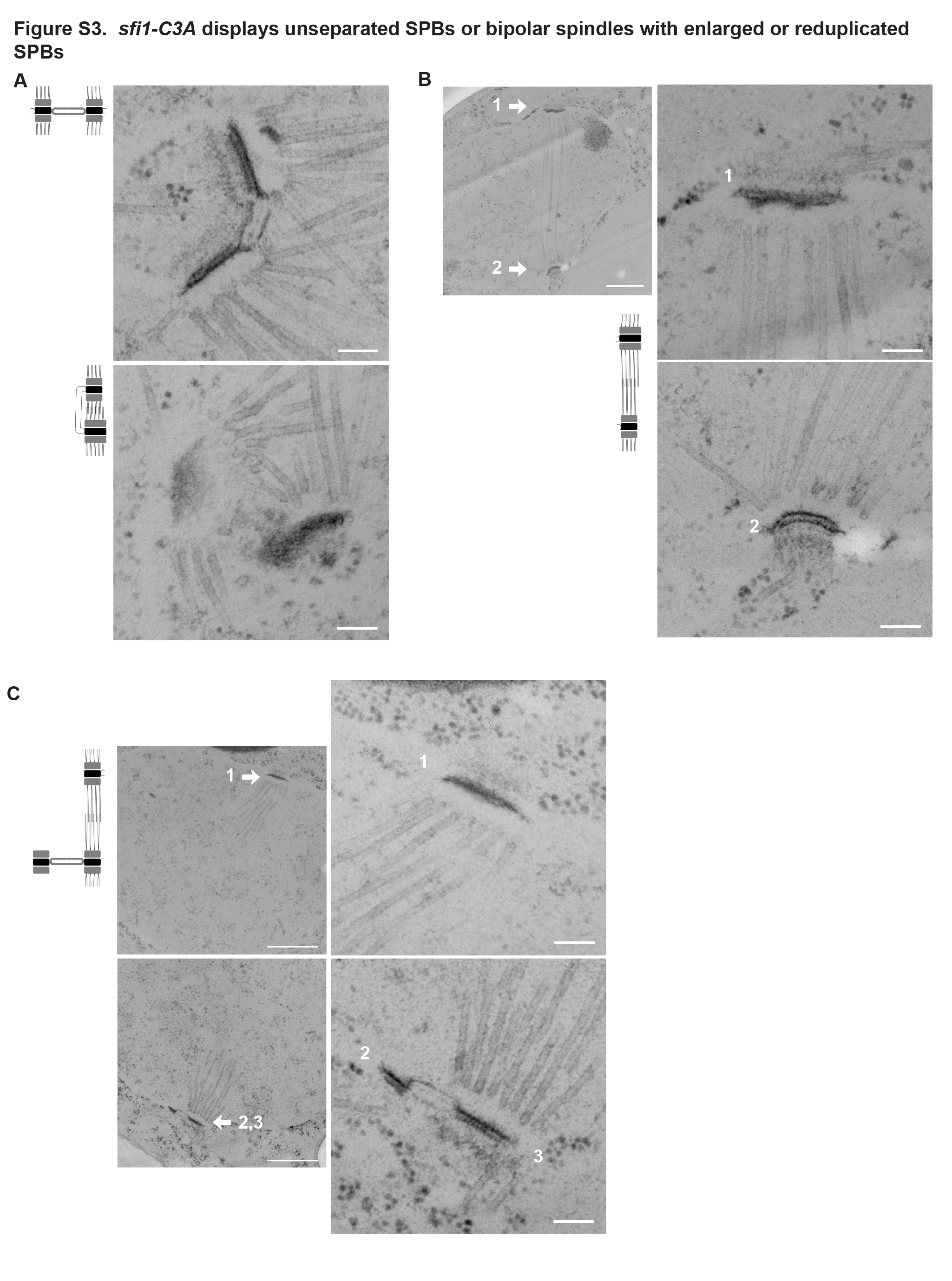

Supplement: Figure S3 — sfi1-C3A displays unseparated SPBs or bipolar spindles with enlarged or reduplicated SPBs. A–C. Asynchronous sfi1-C3A (JA188) cells shifted at early-log phase to 37°C for 4 h in YPD were prepared for EM. Serial sections were examined for 21 cells. A. Representative cells containing two SPBs at one pole with SPBs in a side-by-side configuration (n = 8; upper panel) or SPBs with an abnormal orientation (n = 7; lower panel). Scale bar: 100 nm. B–C. Cells contain short bipolar spindles with aberrant SPBs. Left panels: full spindle, 500 nm scale bar. Right panels: SPB(s), 100 nm scale bar. B. Representative cell (n = 3) with an abnormally large SPB in the upper panel. C. Representative cell (n = 3) containing a short bipolar spindle with three SPBs. The upper panel identifies a single SPB (1), while the lower panel shows a mature SPB (3) connected via a bridge to a partial SPB (2). Average distance between adjacent SPBs: 148±22 nm. (TIF) [file pgen.1004666.s003.tif]

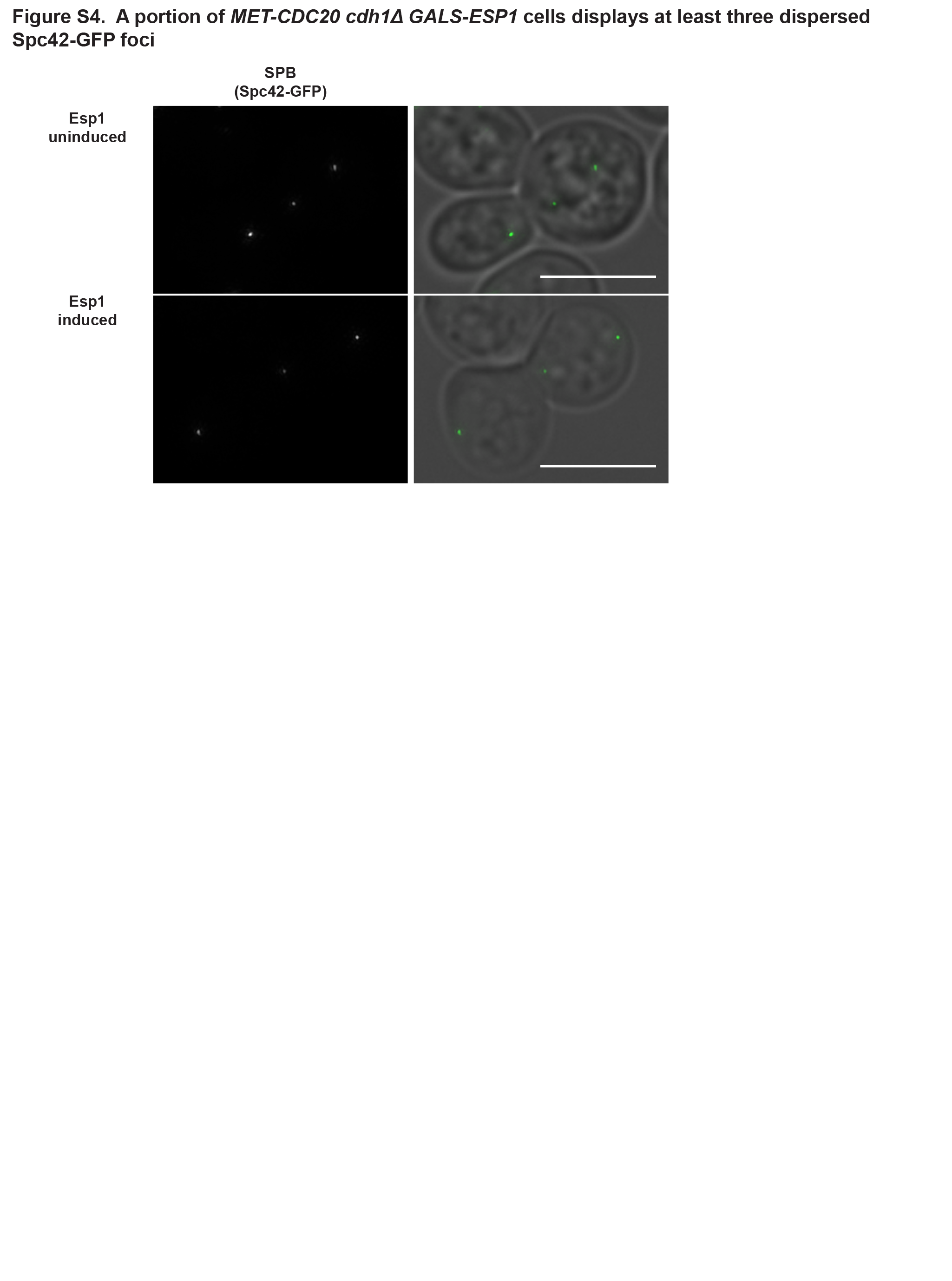

Supplement: Figure S4 — A portion of MET-CDC20 cdh1Δ GALS-ESP1 cells displays at least three dispersed Spc42-GFP foci. An asynchronous culture of MET-CDC20 cdh1Δ GALS-ESP1 SPC42-GFP (JA256) cells was grown to early-log phase in SC-Met with 3% raffinose. Methionine was added (2 mM final concentration) to arrest cells in metaphase and every 2 h for the experiment remainder. After metaphase arrest, Esp1 either was induced for 4 h using 3% galactose to release Cdc14 from the nucleolus or remained uninduced in raffinose. Fixed large-budded cells were imaged by SIM, with GFP on left and merge with transmitted image on right. A portion of cells, both without (25±8%, upper panel) and with (17±7%, lower panel) Esp1 induction, contain at least three dispersed Spc42-GFP foci. Bar: 5 µm. n≥211 per group from 2 experiments. (TIF) [file pgen.1004666.s004.tif]
