# Supplementary material for: Licensing of Yeast Centrosome Duplication Requires Phosphoregulation of Sfi1
Source: PLoS Genet. 2014 Oct 23;10(10):e1004666. doi: 10.1371/journal.pgen.1004666 (PMC4207612; doi:10.1371/journal.pgen.1004666)
Supplement: Table S1 — Yeast strains. Yeast strains used in this study. (DOC) [file pgen.1004666.s005.doc]

**Table S1. Yeast strains**

| **Strain** | **Genotype** |
| --- | --- |
| JA162 | *SFI1-NATMX ADE2 MATa* |
| JA184 | *sfi1-T816A S882A S892A S923A-NATMX ADE2 MATa* |
| JA188 | *sfi1-C3A-NATMX ADE2/sfi1-C3A-NATMX ADE2 MATa/MATa* |
| JA192 | *sfi1-T816A S892A-NATMX ADE2 MATa* |
| JA196 | *SFI1-NATMX ADE2/SFI1-NATMX ADE2 MATa/MATα* |
| JA217 | *sfi1-S855A-NATMX ADE2 MATa* |
| JA249 | *sfi1-C4A-NATMX ADE2/sfi1-C4A-NATMX ADE2 MATa/MATa* |
| JA254 | *SFI1-NATMX SPC42-GFP-KANMX ADE2 MATa* |
| JA256 | *cdc20::MET-CDC20-TRP1 cdh1Δ::LEU2 esp1::GALS-ESP1-URA3 SPC42-GFP-KANMX ADE2 MATα* |
| JA266 | *sfi1-T816A S855A S882A S892A S923A-NATMX ADE2/sfi1-T816A S855A S882A S892A S923A-NATMX ADE2 MATa/MATa* |
| JA295 | *SFI1 leu2::GAL1-pds1-mdb-LEU2 SPC42-GFP-KANMX ADE2 MATa* |
| JA297 | *sfi1-C4A-NATMX leu2::GAL1-pds1-mdb-LEU2 SPC42-GFP-KANMX ADE2 MATa* |
| JA302 | *sfi1-C4A-NATMX SPC42-GFP-KANMX ADE2 MATa* |
| JA308, JA309 | *trp1::GFP-sfi1-C3A-NATMX-TRP1 sfi1Δ::HIS5/trp1::GFP-sfi1-C3A-NATMX-TRP1 sfi1Δ::HIS5 pLEU-HIS-SPC42-mCherry MATa/MATa* |
| JA310, JA311 | *trp1::GFP-SFI1-NATMX-TRP1 sfi1Δ::HIS5/trp1::GFP-SFI1-NATMX-TRP1 sfi1Δ::HIS5 pLEU-HIS-SPC42-mCherry MATa/MATα* |
| JA353, JA354 | *trp1::GFP-sfi1-C4A-NATMX-TRP1 sfi1Δ::HIS5/trp1::GFP-sfi1-C4A-NATMX-TRP1 sfi1Δ::HIS5 pLEU-HIS-SPC42-mCherry MATa/MATa* |
